# A Clinical Mentorship and Quality Improvement Program to Support Health Center Nurses Manage Type 2 Diabetes in Rural Rwanda

**DOI:** 10.1155/2017/2657820

**Published:** 2017-12-03

**Authors:** Aphrodis Ndayisaba, Emmanuel Harerimana, Ryan Borg, Ann C. Miller, Catherine M. Kirk, Katrina Hann, Lisa R. Hirschhorn, Anatole Manzi, Gedeon Ngoga, Symaque Dusabeyezu, Cadet Mutumbira, Tharcisse Mpunga, Patient Ngamije, Fulgence Nkikabahizi, Joel Mubiligi, Simon Pierre Niyonsenga, Charlotte Bavuma, Paul H. Park

**Affiliations:** ^1^Partners in Health/Inshuti Mu Buzima, Kigali, Rwanda; ^2^Ministry of Health, Kigali, Rwanda; ^3^Harvard Medical School, Boston, MA, USA; ^4^Partners in Health, Freetown, Sierra Leone; ^5^Northwestern University Feinberg School of Medicine, Chicago, IL, USA; ^6^Partners in Health, Boston, MA, USA; ^7^University of Rwanda College of Medicine and Health Sciences, Kigali, Rwanda

## Abstract

**Introduction:**

The prevalence of diabetes mellitus is rapidly rising in SSA. Interventions are needed to support the decentralization of services to improve and expand access to care. We describe a clinical mentorship and quality improvement program that connected nurse mentors with nurse mentees to support the decentralization of type 2 diabetes care in rural Rwanda.

**Methods:**

This is a descriptive study. Routinely collected data from patients with type 2 diabetes cared for at rural health center NCD clinics between January 1, 2013 and December 31, 2015, were extracted from EMR system. Data collected as part of the clinical mentorship program were extracted from an electronic database. Summary statistics are reported.

**Results:**

The patient population reflects the rural settings, with low rates of traditional NCD risk factors: 5.6% of patients were current smokers, 11.0% were current consumers of alcohol, and 11.9% were obese. Of 263 observed nurse mentee-patient encounters, mentor and mentee agreed on diagnosis 94.4% of the time. Similarly, agreement levels were high for medication, laboratory exam, and follow-up plans, at 86.3%, 87.1%, and 92.4%, respectively.

**Conclusion:**

Nurses that receive mentorship can adhere to a type 2 diabetes treatment protocol in rural Rwanda primary health care settings.

## 1. Introduction

Diabetes mellitus, along with other noncommunicable diseases (NCDs), was at one time considered rare in low- and middle-income countries (LMICs) [1]. With the recent epidemiological shift in disease burden from infectious to noncommunicable conditions, the prevalence of diabetes is rapidly rising, and now, 80% of the 415 million individuals living with diabetes reside in LMICs [2, 3]. That said, sub-Saharan Africa (SSA) presents with a unique burden of diabetes presentation inclusive of endemic illness among the poorest populations which is commonly undocumented. This mostly rural population with largely agriculture-based communities includes higher rates of underweight patients as opposed to obese. As a result, unique patient presentations related to malnutrition and type 1 diabetes are relatively more common [4]. When looking at national population-based databases for diabetes inclusive of urban areas, SSA is also expected to experience a large increase in disease prevalence [3]. Currently, an estimated 14.2 million adults are living with diabetes in SSA and this number is predicted to increase to 34.2 million adults by the year 2040 [2]. Reported raw regional prevalence within SSA varies widely, between 2.1 and 6.7% [2, 5]. Type 2 diabetes accounts for 90–95% of identified cases in this region [5], and it is estimated that a large portion of cases goes undiagnosed as a result of delays in care-seeking related to late onset of symptoms and challenges presented by lack of resources and government support for early detection and screening [6–9].

In Rwanda, there were approximately 194,300 cases of diabetes diagnosed in adults and about 5000 diabetes-related deaths in 2015 [10, 11]. A retrospective study of patients in three rural districts who enrolled in a NCD program between October 1, 2006 and September 30, 2014 found that 544 patients received treatment for diabetes. The majority of patients had type 2 diabetes, and median baseline glycated hemoglobin (HbA1c) was 10.3% [12]. While Rwanda is one of the few countries in SSA to have a strategic NCD plan integrated into its public health care system that includes diabetes, management of this complex chronic disease is still challenging [7, 10].

Diabetes management requires a health system that is able to detect, diagnose, treat, and provide long-term continuous management for patients. The health systems in many countries of SSA are limited in their capacity to do this [13–15]. The resources needed to effectively manage a chronic disease like diabetes are often clustered in urban areas, with specialists and existing programs primarily available at referral hospitals or targeted toward specific subpopulations [16, 17]. As much of the population of SSA resides in rural areas, the need to establish high-quality service delivery in a more geographically accessible manner is critical. Decentralization of services from the referral or a district hospital level to the health center level is one approach that makes health care more accessible [18]. However, decentralization does not ensure high-quality care delivered by competent providers. In addition to the lack of physical resources, one of the foremost limitations of delivering care is the inadequate number of skilled health care providers [19]. In much of SSA, primary care is delivered at health centers by nurses who are the front-line health care providers and expected to know how to treat a wide variety of diseases [19–21]. Nevertheless, formal classroom and clinical training are not standardized, and continuing nurse education is unavailable or inefficient [22].

Several approaches have been suggested to close competency gaps for health care providers working in primary health care settings in LMICs. However, we have learned from the HIV treatment experience [23] and through additional research that targeted trainings, simplified clinical treatment guidelines, and clinical supervision are not enough to render nurses capable of providing quality care [24]. Instead, robust continuous mentorship and close monitoring are best suited to identify and respond to competency gaps and other system-level issues that obstruct quality care delivery [25]. Such gaps are especially concerning in more remote health facilities where resources, including specialized training, medications, and equipment, are more limited.

In 2010, the Rwanda Ministry of Health (MOH) and the international nonprofit organization Partners In Health/Inshuti Mu Buzima (PIH/IMB) developed an initiative to address competency gaps at the health center level. The Mentorship and Enhanced Supervision for Health Care and Quality Improvement (MESH-QI) program uses mentorship in a clinical setting to support nurses working at health centers to provide quality care to patients and introduce quality improvement initiatives that address systemic issues that hinder health care delivery at this level [26]. This program has supported the delivery of many innovative MOH-PIH/IMB clinical services and shown to be a sustainable initiative that addresses health care provider and system limitations [27–29]. Recently, it was adapted to support the decentralization of NCD services from district hospitals to health centers. In doing so, NCD-specific training and medical supplies were provided to rural health centers with the aim of delivering quality, protocol-driven clinical care by trained nurses.

In this paper, we describe how MESH-QI operates and the quality of MESH-QI supported diabetes care provided in the context of decentralizing NCD services in three rural districts of Rwanda. We also describe the degree of adherence to the type 2 diabetes management protocol of nurses working in health center NCD clinics under this structured clinical mentorship and quality improvement program. We hope our experience offers insight into a strategy that can address care delivery gaps, improve quality of care, and expand access to care for diabetes patients in similar settings.

## 2. Methods

### 2.1. Study Setting

The MOH launched an integrated NCD program in collaboration with PIH/IMB at three rural district hospitals in 2006 [30, 31]. Briefly, the integrated NCD model provides decentralized nurse-led services at rural district hospitals. The delivery platform includes hypertension, diabetes, heart failure, and asthma treatments. Specifically, severe NCDs, such as type 1 diabetes and rheumatic heart disease, are also included as part of the training and mentorship, essential medicines and equipment procurement, and integrated care delivery. This integrated approach provides a resource-efficient model which ensures adequate patient volumes. In response to the growing patient volume and demand for more geographically accessible care, decentralization of the integrated program for common NCDs (hypertension, type 2 diabetes, and asthma) to health centers within the hospitals' catchment areas was part of the initial implementation model at a few sites and then began a rapid scale-up of health center-based delivery in 2012. This study includes the ten health centers that had NCD clinics established in the catchment areas of Rwinkwavu, Kirehe, and Butaro district hospitals at the time of the study period.

Task shifting is an approach used by PIH/IMB and the MOH to support the progressive decentralization of NCD services. Informed by models used to treat HIV in low-resource settings, task shifting in the case of NCDs takes services traditionally provided by physicians and makes them the responsibility of nurses [32–35]. Decentralization has also been facilitated through the use of standardized protocols to guide treatment provision. Diabetes treatment at the MOH-PIH/IMB NCD clinics is guided by the national clinical protocol that was developed by the MOH, PIH/IMB, and specialists from other partner organizations [30]. If an individual presents at a health center clinic with signs and symptoms of type 1 or 2 diabetes, the MOH NCD-trained nurse follows the protocol algorithms to make decisions for diagnosis and treatment or referral. Diagnosis and monitoring use point-of-care testing for urine glucose, urine ketones, blood glucose, and HbA1c. The initial diagnosis is confirmed by a physician at the district hospital NCD clinic. Only patients with controlled, noninsulin-dependent type 2 diabetes are treated at the health center level; all other diabetes patients, including type 1 diabetes, continue to receive follow-up care at the district hospital. The frequency of patient follow-up visits ranges from weekly to every two months depending on severity.

In Rwanda, there are three levels of nurses. Level A2 nurses have a secondary school nursing degree. This cohort makes up the bulk of the health care workforce in Rwanda and most commonly staff the health centers. Level A1 nurses have three years of post secondary training, and level A0 nurses have a bachelor's degree; this cohort typically staffs district hospitals or are in management positions. The health center NCD clinics are staffed by two MOH nurses, usually A2 level, who have received specialty training in the management of NCDs. However, there exists concerns as to whether A2 level nurses can responsibly manage NCD patients in a rural context.

### 2.2. MESH-QI for NCDs

The NCD MESH-QI program was established in 2012 with the objectives of improving the knowledge base and skills of nurses, supporting in-service skill transfer, addressing system gaps such as the supply chain, and sustaining improvements in NCD care delivery at the health center level. At each district hospital, the MOH nurse who had the most advanced training and experience among the two NCD nurses was selected to receive training in mentorship and quality improvement techniques for one week. At the time of this study, the nurse mentor from Kirehe District Hospital was level A2 and had more than five years of experience treating NCDs, the nurse mentor from Rwinkwavu District Hospital was level A1 and had two years of NCD experience, and the nurse mentor from Butaro District Hospital was level A0. Per the program design, the nurse mentors aimed to visit each health center NCD clinic once every four to six weeks; during which time, they would pair with the NCD nurse(s) (referred to as “nurse mentees”) and observe mentee-patient consultations to provide real-time feedback on care provision.

The key tool of the MESH-QI program is a structured observation checklist that is used to assess nurse mentees' adherence to the disease management protocol and provide a measure of quality of care [26]. The diabetes checklist mirrors the diabetes management protocol algorithms and lists key assessment tasks the nurse mentee should be performing during his or her consultation with each patient. Similar to the protocol, the checklist is organized by a type of patient visit (first visit versus follow-up visit) and a type of clinical task (e.g., patient history, physical exam, treatment and management, and counseling). For certain tasks, the checklist prompts the mentor to record if the nurse mentee properly documented the clinical information in the patient's chart. The section on treatment and management involves more clinically advanced skills (diagnosis, referral, medication management, hypoglycemia management, and follow-up plan) and prompts the nurse mentor to indicate whether she/he agrees with the action taken by the nurse mentor. One checklist is manually completed by the nurse mentor for each nurse mentee-patient consultation they observe during their health center visit.

### 2.3. Study Design and Population

We describe the demographics and diabetes risk factors of type 2 diabetes patients enrolled in the MOH-PIH/IMB NCD program and followed at NCD health center clinics between January 1, 2013 and December 31, 2015. We also summarize the results of the MESH-QI checklists completed by MESH NCD nurse mentors during their observations of nurse mentee-patient consultations between January 1, 2013 and December 31, 2015.

### 2.4. Data Sources and Statistical Analysis

Routinely collected patient information was recorded on diabetes-specific paper charts, which were then entered into OpenMRS, the electronic medical record (EMR) system utilized by PIH/IMB. Missing or inaccurate values were reconciled as needed. Baseline demographic and risk factor data from type 2 diabetes patients who enrolled in the NCD program during the study window were extracted from the EMR. Risk factor data included key variables known to be risk factors for diabetes, that is, body mass index, tobacco, and alcohol use and education level. Descriptive statistics were calculated. Median and interquartile ranges were reported for continuous variables and frequencies, and percentages were reported for categorical data.

Starting in 2013, nurse mentor observation checklists were entered into a Microsoft Access database managed by PIH/IMB's Monitoring and Evaluation (M&E) Department. M&E coordinators ensured the checklists were complete and correctly entered through routine data quality assessments. Data on the level of training of the nurses working at the NCD clinics, types of patient consultations, adherence of nurse mentees to the diabetes management protocol, and the agreement between mentor and mentee on key aspects on care were extracted from the MESH-QI database.

The checklist data were reported from two different perspectives. First, data was summarized by a clinic day. A clinic day was defined as one nurse mentor's visit to one health center NCD clinic. Due to the design of the NCD clinic, it was likely that the mentor observed the same nurse mentee perform multiple patient consultations during his visit. Therefore, visit and nurse-related information was reported by this parameter. Data was also summarized by nurse mentee-patient consultation. Our other outcomes of interest, which include nurse mentee adherence to the diabetes protocol and mentor-mentee agreement on diabetes treatment plans for each observed patient, are reported per nurse mentee-patient consultation. “Adherence” was defined as the proportion of protocol-driven assessment tasks completed by nurse mentees during patient encounters. “Mentor-mentee agreement” was defined as the proportion of consultations in which the nurse mentee correctly assessed patient illness, treatment, and medication (using mentor classifications as the standard and thusly measuring whether mentor agrees with mentee assessment). Stata v14 (StataCorp, College Station, Texas, US) was used for data cleaning and analysis.

### 2.5. Ethical Considerations

This study was approved by the Rwanda National Health Research Committee, the Rwanda National Ethics Committee, and the Partners Health Care Institutional Review Board in Boston, Massachusetts, USA. This study used routinely collected programmatic data that were deidentified; therefore, consent to participate was not applicable.

## 3. Results

Table 1 provides an overview of the profile of diabetes patients in the PIH/IMB-supported districts of rural Rwanda. Demographic and clinical characteristics of the 128 type 2 diabetes patients enrolled in care from January 1, 2013 to December 31, 2015, are reported. Median age was 54 (IQR = 42, 61), and 63.3% (*N* = 81) were female. Few patients (5.6%, *N* = 7 of 124) were current smokers or consumers of alcohol (11.0%, *N* = 13 of 118). Of the 77 patients that had their body mass index (BMI; kg/m^2^) calculated, 2.6% (*N* = 2) were underweight (BMI<18.5), 50.6% (*N* = 39) were normal weight (18.5≥ BMI <25), 35.1% (*N* = 27) were overweight (25≥BMI < 30), and 11.7% (*N* = 9) were obese (BMI≥30). Approximately, half (49.6%, *N* = 36 of 74) of the patients had a primary school education and 74.6% (*N* = 91 of 122) were farmers.

Of the 133 clinic days attended by mentors during the study period, 87.2% (*N* = 116) were staffed by nurses who had been trained in NCD care while the remaining clinic days were run by nurses with no specialized training (Table 2). The total number of observed nurse mentee-patient consultations was 263 (Table 3); 6.8% (*N* = 18) of the observations were of a patient's first visit to the health center while 88.6% (*N* = 233) of the observations were undertaken during patient follow-up visits; the type of visit was not specified for 4.6% (*N* = 12) of the observations. The mentors conducted 39.9% (*N* = 105), 38.0% (*N* = 100), and 22.1% (*N* = 58) of their observations in Burera, Kirehe, and Southern Kayonza districts, respectively.

Adherence of nurse mentees to the type 2 diabetes management protocol is described in Table 4. Of the 18 mentor observations conducted during a patient's first visit to the health center, nurse mentees assessed key risk factors (defined here as family history of diabetes, smoking history, and alcohol use) in 55.5% (*N* = 10), 61.1% (*N* = 11), and 77.8% *N* = 14) of the consultations, respectively. Nurse mentees assessed HIV status in 72.2% (*N* = 13) of the consultations but only documented the HIV status in the patient's medical chart in 11.1% (*N* = 2) of consultations. New patients were asked by mentees about medication use 61.1% (*N* = 11) of the time and for their age at first diagnosis 77.8% (*N* = 14) of the time. Nurse mentees asked for address and phone number 83.3% (*N* = 15) of the time but documented this information only 11.1% (*N* = 2) of the time.

For all patient visits (*N* = 263), nurse mentees should assess symptoms and disease-related complications. Mentees assessed new symptoms in 86.7% (*N* = 228) of the consultations, hypoglycemia symptoms in 81.8% (*N* = 215), peripheral neuropathy symptoms in 77.2% (*N* = 203), missed medications in 82.9% (*N* = 218), and recent hospitalizations in 78.3% (*N* = 206) of the consultations. Blood pressure was both checked and documented in 95.8% (*N* = 252) of consultations. Mentors observed that nurse mentees checked patient weight 93.5% (*N* = 246) of the time and documented weight 11.4% (*N* = 30) of the time. Height was assessed 13.3% (*N* = 35) of the time and documented 11.4% (*N* = 30) of the time. Body mass index was calculated in 14.4% (*N* = 38) of the consultations. Mentees checked patients for foot sores 71.9% (*N* = 189) of the time, and blood sugar was checked in 93.2% (*N* = 245) of encounters. Mentees provided counselling to the patient about their disease, danger signs, and medication use in 86.7% (*N* = 228), 79.1 (*N* = 208), and 44.5% (*N* = 117) of encounters, respectively.

Mentors and mentees agreed on key aspects of type 2 diabetes care for patients attending the NCD clinic for the first time and for those attending the clinic for the follow-up visits (Table 5). For first patient visits (*N* = 18), the mentor denoted agreement with the mentee on diagnosis 94.4% (*N* = 17) of the time and decision regarding the patient's referral to district hospital 88.9% (*N* = 16) of the time. For all visits (*N* = 263), mentors indicated they agreed with the mentees on medication management (86.3%, *N* = 227), hyperglycemia emergency treatment (63.5%, *N* = 167), laboratory exam requests (87.1%, *N* = 229), and follow-up plan (92.4%, *N* = 243) in the majority of patient encounters.

## 4. Discussion

Overall, our study findings suggest that NCD health center nurses provided with training and ongoing mentoring can adhere to the type 2 diabetes treatment protocol in the majority of the patient encounters. These findings are consistent with those from another study which reported strong adherence to diabetes management protocols following training and mentorship interventions (89% for weight check and 93% for blood pressure check) in an urban Kenyan setting [36]. This suggests that training combined with mentorship for primary health care workers may be an effective way to ensure quality while decentralizing type 2 diabetes care using structured treatment protocols. Agreement between mentors and mentees on key aspects of care was evident in diagnosis made and decisions on referral to district hospitals, medication management, laboratory tests requests, and follow-up plan. While this demonstrates that the nurses are largely following the type 2 diabetes management protocol, we cannot yet distinguish the impact mentorship plays independent of the initial training. Adherence to treatment protocols by trained nurses who received posttraining mentorship was also found in other studies [37–39]. However, within our context, it is important to note that nurse mentors did not often agree with how nurse mentees managed hyperglycemia emergencies. Tasks which require more advanced management decision-making like this did not show nurse mentor-mentee agreement levels as high as tasks requiring more routine medication decisions. This shows that there are some limitations to protocol-driven treatment, and this should be further explored. Advanced decision-making and problem-solving should be an area that nurse mentors focus on during their interactions with the nurse mentees.

While nurses trained in NCDs plus HIV or mental health care (or all 3 areas) conducted some consultations, we found that the health center NCD clinics were run by nurses specially trained only in NCD care in the majority of the clinic days. While we expected this outcome, we are highlighting it here to point to the fact that the health centers with NCD clinics had the proper human resource systems in place, and with the support of nurse mentors, staffs were able to appropriately organize services such that NCD-trained nurses were in the clinic routinely.

Despite the findings that nurses are following the type 2 diabetes treatment protocol, there is still a room for improvement with regard to the documentation of clinical assessment findings by nurses, which is essential for continuity of care, follow-up management, and the management of chronic conditions. The importance of proper documentation and counselling on medication should be emphasized by NCD mentors during routine mentorship visits. In addition, counselling on medication could be improved. These gaps could be addressed through the NCD mentors during routine mentorship visits.

The demographic profile of the type 2 diabetes patients included in our study is illustrative of the rural setting of the catchment area, especially with regard to education level and occupation. As expected, tobacco use, alcohol use, and obesity, the known typical urban-based risk factors of NCDs, including type 2 diabetes, were not common characteristics found in our mostly rural study population [40]. These findings support the idea that the widely accepted behavioral risk factor model for NCDs is not widely applicable to rural populations [41]. Further investigation into the risk factors unique to rural populations could improve disease diagnosis and management.

Our study had a number of limitations. Missing demographic and clinical data from the EMR system and observation checklists narrowed the scope of our analysis. Data on clinical outcomes of the patients included in the study sample were not extracted so we are unable to make any conclusions about diabetes control or retention in the sample. Also, relying on performance measurements collected by the mentors themselves may have introduced bias in their assessment of nurse mentees' performance. Similarly, due to the Hawthorne effect, nurse mentees may have displayed best practices only in the presence of a mentor which may lead to an overestimate of the degree of protocol adherence [42]. However, the mentorship model's emphasis on relationship building and repeated mentorship observations may have reduced this effect. While we have no data on the nature of the mentor-mentee interactions and it is not possible to draw direct cause and effect of the nurse mentors' observations and support on the performance of the nurse mentees, our findings still support the feasibility of mentorship as an approach for bridging the know-do gap and supporting the delivery of care provided by primary health care nurses.

Though our study findings suggest that MESH-QI can be an effective approach to decentralize type 2 diabetes care in rural primary healthcare settings, these results should be interpreted with caution. The lack of pre-MESH-QI type 2 diabetes care delivery data did not allow us to determine the level of changes after the intervention. As such, other factors may have contributed to reported improvements. Furthermore, the lack of control group and patient level outcome measurements limited our ability to determine the impact of the MESH-QI intervention on patient outcomes. Further rigorous evaluative studies are needed to explore the impact of this mentorship and quality improvement initiative on patients' clinical outcomes and retention in care ahead of the program being rolled out in other settings. Also, determining this model's effectiveness in the context of scale-up beyond the three PIH/IMB-supported districts will provide valuable insights to inform future healthcare delivery policies.

## 5. Conclusions

The results from our study suggest that specific NCD training of health center nurses complemented by clinical mentorship and quality improvement initiatives can result in nurse adherence to diabetes management protocols. This suggests that type 2 diabetes patients are receiving high-quality care; the monitoring and reporting of which were possible through the MESH-QI mentors' systematic documentation of mentorship and care provision. Clinical mentorship is a feasible model to address challenges to service delivery in rural areas. This approach can be used to progressively decentralize NCD services from district hospitals to health centers in other districts of Rwanda as well as in similar resource-limited settings.

## Figures and Tables

**Table 1:** Demographic and clinical characteristics of type 2 diabetes patients enrolled in a noncommunicable disease program (January 1, 2013–December 31, 2015).

Total number of patients	*N* = 128	
	*N*	%
Age in years (median, IQR^∗^)	54	42, 61
Gender		
Male	47	36.7
Female	81	63.3
District of residence		
Burera	67	52.3
Kayonza	13	10.2
Kirehe	33	25.8
Other	15	11.7
Education level (*N* = 74)		
Primary	36	48.6
Secondary and above	19	25.7
None	19	25.7
Occupation (*N* = 122)		
Driver	3	2.5
Farmer	91	74.6
Other employed	22	18.0
Retired	2	1.6
Unemployed	3	2.5
Child or adolescent	1	0.8
Tobacco use (*N* = 124)		
Current smoker	7	5.6
Past smoker	21	16.9
Never smoked	96	77.4
Alcohol use (*N* = 118)		
Current	13	11.0
Past	37	31.4
Never	68	57.6
HIV status (*N* = 121)		
Positive	5	4.1
Negative	89	73.6
Patient does not know	27	22.3
Body mass index (kg/m^2^) (*N* = 77)		
Underweight (<18)	2	2.6
Normal weight (18.5 ≥ BMI< 25)	39	50.6
Overweight (25 ≥ BMI< 30)	27	35.1
Obese (≥30)	9	11.7
Location of first health facility encounter		
Butaro hospital	25	19.5
NCD clinics at health centers in Burera District	3	2.3
Kirehe District Hospital	51	39.8
NCD clinics at health centers in Kirehe District	16	12.5
Rwinkwavu District Hospital	18	14.1
NCD clinics at health centers in Southern Kayonza District	15	11.7

^∗^IQR: interquartile range.

**Table 2:** Specialized fields of training of nurse mentees observed (January 1, 2013–December 31, 2015).

Number of clinic days when observations were done	*N* = 133	
	*N*	%
Field of training		
No specialized training	17	12.8
NCD^∗^ training only	84	63.2
HIV training only	0	0.0
NCD & HIV training	26	19.5
NCD & MH^±^ training	2	1.5
NCD & HIV & MH	4	3.0

^∗^NCD: noncommunicable disease; ^±^MH: mental health.

**Table 3:** Mentor observations of nurse mentee-patient encounters (January 1, 2013–December 31, 2015).

Total number of mentor observations	*N* = 263	
	*N*	%
Type of encounter observed		
Patient first visit	18	6.8
Patient follow-up visit	233	88.6
Not documented	12	4.6
District where observation was conducted		
Southern Kayonza	58	22.1
Kirehe	100	38.0
Burera	105	39.9

**Table 4:** Adherence of nurse mentees to the diabetes management protocol (January 1, 2013–December 31, 2015).

Asked only at first patient visit	*N* = 18	
	*N*	%

*Patient history*		
Mentee asked for family history of diabetes		
Yes	10	55.5
No	5	27.8
Missing	3	16.7
Mentee asked about smoking history		
Yes	11	61.1
No	1	5.6
Missing	4	22.2
Question not applicable (patient < 15 years)	2	11.1
Mentee asked about alcohol use		
Yes	14	77.8
No	1	5.5
Missing	3	16.7
Mentee asked for HIV status		
Yes	13	72.2
No	2	11.1
Missing	3	16.7
Mentee documented HIV status		
Yes	2	11.1
No	16	88.9
Mentee asked about current medication use		
Yes	11	61.1
No	4	22.2
Missing	3	16.7
Mentee asked for age at first diagnosis		
Yes	14	77.8
No	2	14.3
Missing	2	14.3
Mentee asked for phone number		
Yes	15	83.3
No	0	0.0
Missing	3	16.7
Mentee documented phone number		
Yes	2	11.1
No	16	88.9
Mentee asked for address		
Yes	15	83.3
No	0	0.0
Missing	3	16.7
Mentee documented address		
Yes	2	11.1
No	16	88.9
Mentee asked for national identification number		
Yes	6	33.3
No	6	33.3
Missing	6	33.3
Mentee documented national identification number		
Yes	3	16.7
No	1	5.5
Missing	14	77.8

Asked at all patient visits	*N* = 263	
	*N*	%

*Patient history*		
Mentee asked about new symptoms		
Yes	228	86.7
No	26	9.9
Missing	9	3.4
Mentee asked about hypoglycemia symptoms		
Yes	215	81.8
No	38	14.4
Missing	10	3.8
Mentee asked about peripheral neuropathy		
Yes	203	77.2
No	48	18.2
Missing	12	4.6
Mentee asked about missed medications		
Yes	218	82.9
No	23	8.7
Missing	15	5.7
Not applicable (patient not on regularly scheduled medications)	7	2.7
Mentee asked about recent hospitalizations		
Yes	206	78.3
No	43	16.3
Missing	14	5.3
Mentee asked about pregnancy (for female patients only; *N* = 114)		
Yes	93	81.6
No	13	11.4
Missing	8	7.0
*Physical exam*		
Mentee checked blood pressure		
Yes	252	95.8
No	1	0.4
Missing	10	3.8
Mentee documented blood pressure		
Yes	252	95.8
No	2	0.8
Missing	9	3.4
Mentee checked height		
Yes	35	13.3
No	2	0.8
Missing	22	8.4
Not applicable (patient > 18 years)	204	77.5
Mentee documented height		
Yes	30	11.4
No	2	0.8
Missing	21	8.0
Not applicable (patient > 18 years)	210	79.8
Mentee checked weight		
Yes	246	93.5
No	6	2.3
Missing	11	4.2
Mentee documented weight		
Yes	30	11.4
No	2	0.8
Missing	231	87.8
Mentee calculated patient's body mass index		
Yes	38	14.4
No	225	85.6
Mentee checked for foot sores		
Yes	189	71.9
No	59	22.4
Missing	15	5.7
Mentee checked blood sugar		
Yes	245	93.2
No	8	3.0
Missing	10	3.8
Mentee checked patient's heart and lungs with stethoscope		
Yes	52	19.8
No	14	5.3
Missing	34	12.9
Not applicable (no dyspnea, chest pain, or leg edema)	163	62.0
Mentee documented patient's heart and lungs with stethoscope		
Yes	56	21.3
No	8	3.0
Missing	35	13.3
Not applicable (no dyspnea, chest pain, or leg edema)	164	62.4
*Counseling and teaching*		
Mentee provided disease counseling		
Yes	228	86.7
No	30	11.4
Missing	5	1.9
Mentee provided danger sign counseling		
Yes	208	79.1
No	46	17.5
Missing	9	3.4
Mentee provided medication counseling		
Yes	117	44.5
No	18	6.8
Missing	128	48.7

**Table 5:** Mentor and mentee agreement on diagnosis, treatment, and follow-up plan (January 1, 2013–December 31, 2015).

First patient visit only	*N* = 18	
	*N*	%
Mentor and mentee agree on diagnosis		
Yes	17	94.4
No	1	5.6
Mentor and mentee agree on referral to district hospital		
Yes	16	88.9
No	1	5.6
Missing	1	5.5
For all visits	*N* = 263	
Mentor and mentee agree on how to manage medication		
Yes	227	86.3
No	25	9.5
Missing	11	4.2
Mentor and mentee agree on hyperglycemia emergency treatment (for patients showing signs of emergency)		
Yes	167	63.5
No	6	2.3
Missing	90	34.2
Mentor and mentee agree on lab requests		
Yes	229	87.1
No	21	8.0
Missing	13	4.9
Mentor and mentee agree on follow-up plan		
Yes	243	92.4
No	9	3.4
Missing	11	4.2
Mentee communicated next follow-up visit to patient		
Yes	220	83.7
No	6	2.3
Missing	19	7.2
Not applicable	18	6.8

## Abbreviations

BMI: Body mass index
EMR: Electronic medical record
HbA1c: Glycated hemoglobin
HIV: Human immunodeficiency virus
IQR: Interquartile range
LMIC: Low- and middle-income countries
M&E: Monitoring and evaluation
MESH-QI: Mentorship and Enhanced Supervision for Health Care and Quality Improvement
MOH: Ministry of Health
NCD: Noncommunicable disease
SSA: Sub-Saharan Africa
PIH/IMB: Partners In Health/Inshuti Mu Buzima.
